# Sales of macrolides, lincosamides, streptogramins, and amoxicillin/clavulanate in the in- and outpatient setting in 10 European countries, 2007–2010

**DOI:** 10.1186/s40064-015-1398-4

**Published:** 2015-10-15

**Authors:** Pili Ferrer, Mònica Sabaté, Elena Ballarín, Joan Fortuny, Marietta Rottenkolber, Sven Schmiedl, Joan-Ramon Laporte, Luisa Ibáñez

**Affiliations:** Fundació Institut Català de Farmacologia, Pg Vall d’Hebron 119-129, 08029 Barcelona, Spain; Department of Clinical Pharmacology, University Hospital Vall d’Hebron, Pg Vall d’Hebron 119-129, 08029 Barcelona, Spain; Department of Pharmacology, Toxicology and Clinical Therapeutics, Hospital Universitari Vall d’ Hebron, Univ Autònoma de Barcelona, Bellaterra (Cerdanyola del Vallès), 08193 Barcelona, Spain; DS&E, Global Clinical Epidemiology, Novartis Farmaceutica S.A., Gran Via Corts Catalanes 764, 08013 Barcelona, Spain; RTI Health Solutions, Barcelona, Spain; Institute for Medical Information Sciences, Biometry and Epidemiology, Ludwig-Maximilians Universitaet- Muenchen, Marchioninistrasse 15, 81377 Munich, Germany; Diabetes Research Group, Medical Department 4, University Hospital Munich, Campus Innenstadt, Ludwig-Maximilians-Universität, Ziemssenstr 1, 80336 Munich, Germany; Philipp Klee-Institute for Clinical Pharmacology, Helios Klinik Wuppertal, Heusnerstrasse 40, 42283 Wuppertal, Germany; Department of Clinical Pharmacology, School of Medicine, Faculty of Health, Witten-Herdecke University, Alfred Herrhausen-Strasse 50, 58448 Witten, Germany

**Keywords:** Drug utilisation, Cross-national comparison, Defined daily doses, Prescription rates, Antibacterials, Hospital, Ambulatory, Indication

## Abstract

Monitoring the use of antibiotics is relevant due to the public health impact of microbial resistance, adverse effects, and costs. We present data on the consumption of macrolides, lincosamides, streptogramins and amoxicillin/clavulanate (AMC) between 2007 and 2010 in the in-and outpatient healthcare setting in 10 European countries provided by IMS Health. Antibiotics were classified according to the anatomical therapeutic chemical classification and consumption was expressed in defined daily doses/1000 inhabitants/day (DIDs). We analysed the number of prescriptions by diagnostic codes between 2008 and 2010, based on the International Classification of Diseases, 10th revision (ICD-10). These ICD-10 codes were grouped into four main categories: respiratory infections, genitourinary infections, other infections and other diagnoses. In 2010, the consumption of macrolides and lincosamides ranged from 0.45 DIDs (Sweden) to 5.46 DIDs (Italy), and from 0.04 DIDs (Denmark) to 1.00 DID (Germany), respectively. Streptogramins were available in France, Germany, Italy, Norway, Spain and United Kingdom with a consumption of <0.001 DID exclusively in the hospital setting. The consumption of AMC ranged from <0.001 DIDs (Norway) to 11.67 DIDs (Spain). During the study period, the consumption of macrolides decreased, the consumption of AMC increased in most of European countries, and lincosamides varied very slightly. Macrolides and AMC were mainly prescribed for respiratory infections in all countries but United Kingdom, where most of the prescriptions were assigned to diagnostic codes not clearly related with an infection. Lincosamides were prescribed for the respiratory infections and other infections groups. There was a wide inter-country variability in the percentage of the prescriptions assigned to each of the diagnostic categories. The inter-country differences in the consumption of these antibiotics and their prescription by diagnostic categories point to an inappropriate use of antibiotics.

**Electronic supplementary material:**

The online version of this article (doi:10.1186/s40064-015-1398-4) contains supplementary material, which is available to authorized users.

## Background

Monitoring the use of antibiotics is relevant due to the public health impact of microbial resistance, adverse effects, and costs. Several studies have shown wide variability in the consumption of antibiotics across Europe (Coenen et al. 2009; Elseviers et al. 2007). This study was designed to compare and analyse the use of macrolides, lincosamides, streptogramins, and amoxicillin/clavulanate (AMC) in the in- and outpatient setting across 10 European countries between 2007 and 2010, and to assess their indication for use. This study is part of the PROTECT project (“Pharmacoepidemiological Research on Outcomes of Therapeutics by a European Consortium”, www.imi-protect.eu), which is a collaborative European project aimed at developing, testing and disseminating methodological standards for the design, conduct and analysis of pharmacoepidemiological studies.

## Results

Macrolides, lincosamides, and AMC were more used in ambulatory care than they were in the hospital setting over the entire study period in all countries. In 2010, the proportion of use of macrolides and lincosamides in hospitals out of the total use of macrolides and lincosamides ranged from 2.8 % in Norway to 15.0 % in the UK. For AMC, this proportion ranged between 5.1 % in Italy and 41.0 % in the UK. The consumption of streptogramins occurred exclusively in the hospital setting.

In 2010, the overall consumption of macrolides ranged from 0.45 DIDs in Sweden to 5.46 DIDs in Italy. In France, Norway, Poland, Spain, and Sweden, the consumption of macrolides continuously decreased over the study period. In Denmark, Germany, Italy, and the UK, the consumption of macrolides increased from 1.8 % in Italy to 6.7 % in the UK, from 2007 to 2010. In the Netherlands, the use of macrolides remained stable (1.46 DIDs). Clarithromycin, azithromycin and erythromycin were the primary macrolides consumed in all countries.

The consumption of lincosamides remained stable or decreased slightly over the study period in all countries. Clindamycin was the primary lincosamine consumed, except in Italy, where the most used was lincomycin. Because of the low consumption rate of streptogramins (<0.0001 DIDs), the results for this group of antibiotics will not be discussed further.

**Fig. 1:**
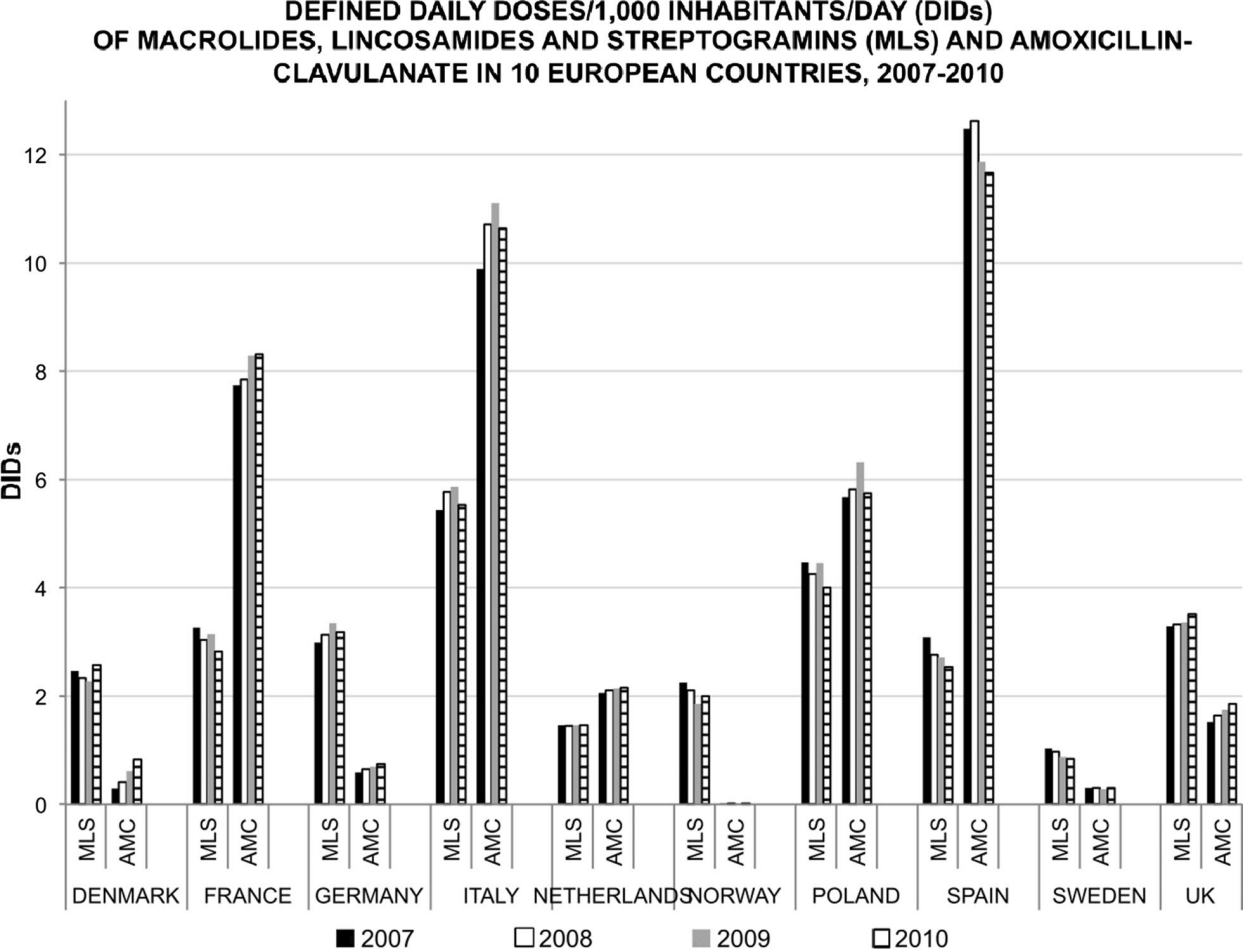
Use of macrolides, lincosamides and stretograpmins (MLS) and amoxicillin clavulanate in 10 European countries, 2007–2010

From 2007 to 2010, the consumption of AMC increased in most countries, especially in Germany (27.6 %) and the UK (22.4 %). However, it decreased in Spain by 6.4 % and remained stable in Sweden (0.30 DIDs). See Fig. 1 for a description of the volume of macrolides, lincosamides, streptogramins (MLS) and AMC. See Additional file 1 for a figure describing erythromycin, clarithromycin, azithromycin and other macrolides use between 2007 and 2010, by country. Additional file 2 supplements the information of use of these antibacterial drugs presenting figures of their prescription rates by country between 2008 and 2010.

The consumption of macrolides, lincosamides and streptogramins has been presented as a single group of antibacterials compared with the consumption of amoxicillin-clavulanate by country, expressed in defined daily doses/1000 inhabitants/day over the study period.

MLS were prescribed for 1045 unique diagnostic codes and AMC was prescribed for 989 different diagnostic codes. Prescription rates showed that all the antibiotics included in this study were mainly prescribed for respiratory infections with slight variations over the study period that did not change the overall pattern. When looking at more detailed diagnostic categories related to infections over all the study period, macrolides were mainly used for bronchitis, pharyngitis, and gastrointestinal infections. Lincosamides were mainly prescribed for tonsillitis, gastrointestinal infections, and skin infections. Streptogramins were mainly prescribed for bronchitis, sinusitis and skin infections. AMC was mainly used for tonsillitis, bronchitis, otitis media and gastrointestinal infections.

**Fig. 2:**
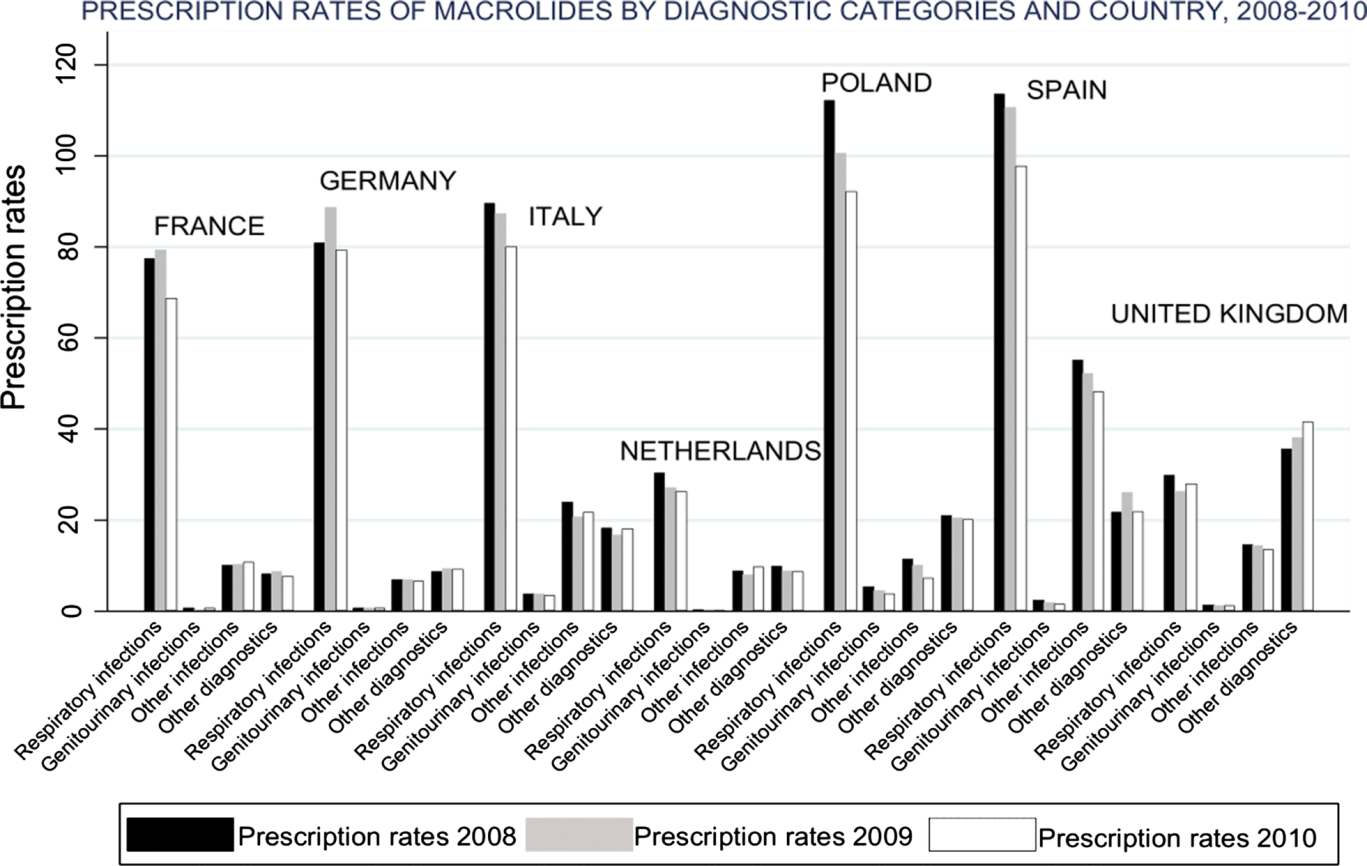
Prescription rates (×1000) of macrolides by diagnostic category and country, 2008–2010

**Fig. 3:**
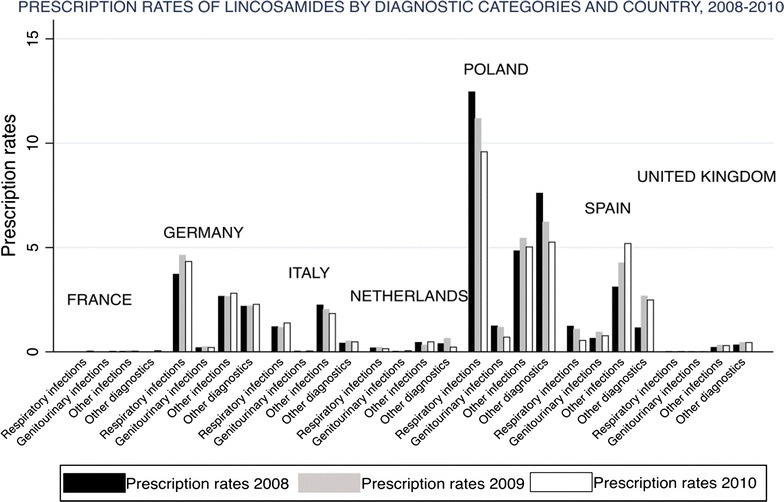
Prescription rates (×1000) of lincosamides by diagnostic categories and country, 2008–2010

All countries indicated the use of macrolides for respiratory infections, with a proportion that varied between 33.2 % in United Kingdom and 85.1 % in France. In United Kingdom, macrolides were mainly prescribed for the other diagnoses group. France, Netherlands, Spain and the United Kingdom used lincosamides for other diagnoses group, whereas in Germany and Poland were mainly used for respiratory infections. In Italy, approximately 50 % of the prescriptions of lincosamides were used for other infections. See Figs. 2 and 3 showing the prescription rates of macrolides and lincosamides, respectively, by diagnostic category and country. See Additional files 2 and 3 showing a figure of the prescription rates of erythromycin, clarithromycin, azithromycin and other macrolides by diagnostic category and country.

**Fig. 4:**
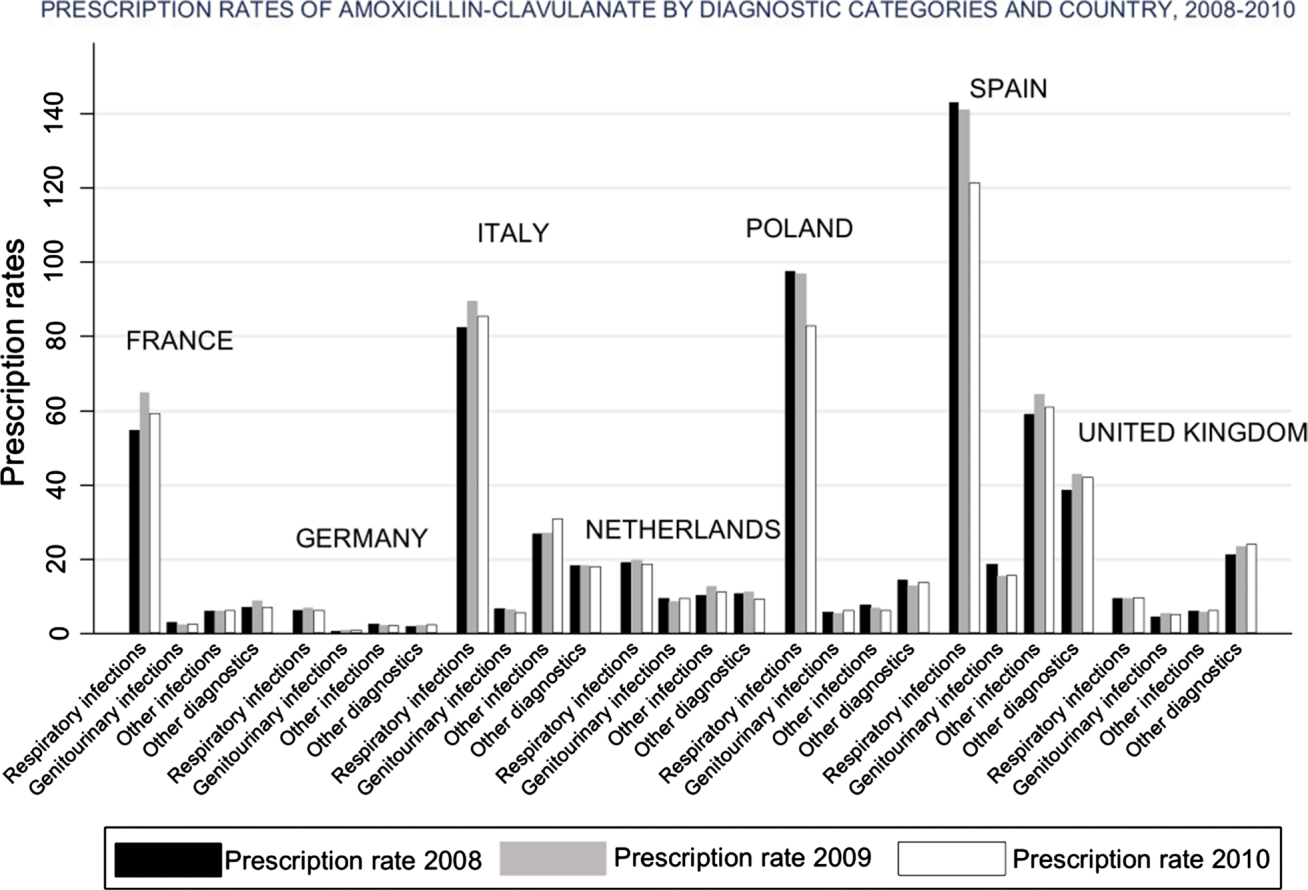
Prescription rates (×1000) of amoxicillin-clavulanate by diagnostic category and country, 2008–2010

For AMC, the pattern of use was similar to that of macrolides over the study period. All countries except the United Kingdom used AMC for respiratory infections, whereas in the United Kingdom 53.5 % of the AMC prescriptions were for other diagnoses. The percentage of use of AMC for respiratory infections ranged between 38.7 % in the Netherlands and 78.9 % in France. Bronchitis, tonsilitis and otitis media were the main indications for use. Figure 4 shows the prescription rates of AMC by diagnostic category and country, 2008–2010.

Tables 1 and 2 show the percentage distribution of the main 10 diagnostic categories for which macrolides and AMC were prescribed for, respectively.

**Table 1:** Distribution of the prescription rates (×1000) of macrolides by country and the 10 most frequently assigned diagnostic categories in 2010

Prescription rates (%)	France	Germany	Italy	Netherlands	Poland	Spain	United Kingdom
Bronchitis	24.6 (28.0)	34.9 (36.4)	21.3 (17.3)	5.1 (11.4)	30.4 (24.7)	17.8 (10.5)	3.6 (4.3)
Pharyngitis	19.4 (22.1)	6.6 (6.9)	17.3 (14.0)	1.1 (2.4)	15.2 (12.3)	23.2 (13.7)	1.3 (1.5)
Gastrointestinal infections	7.9 (9.0)	1.4 (1.5)	10.4 (8.4)	0.6 (1.3)	1.7 (1.4)	41.9 (24.7)	1.1 (1.3)
Other upper respiratory tract infections	4.4 (5.0)	10.9 (11.4)	8.0 (6.5)	3.4 (7.6)	6.6 (5.3)	10.4 (6.1)	4.6 (5.4)
Tonsillitis	0.3 (0.3)	7.3 (7.6)	11.1 (9.0)	2.3 (5.1)	5.5 (4.5)	16.3 (9.6)	1.5 (1.8)
Sinusitis	3.7 (4.2)	8.2 (8.6)	2.6 (2.1)	6.3 (14.0)	9.9 (8.0)	5.1 (3.0)	1.3 (1.5)
Skin infections	1.0 (1.1)	1.6 (1.7)	7.8 (6.3)	6.9 (15.4)	2.7 (2.2)	2.1 (1.2)	10.8 (12.8)
Cold/influenza	6.8 (7.8)	1.1 (1.1)	1.2 (1.0)	0.6 (1.3)	3.2 (2.6)	5.9 (3.5)	0.7 (0.8)
Otitis media	0.8 (0.9)	2.7 (2.8)	3.4 (2.8)	1.9 (4.2)	4.2 (3.4)	6.9 (4.1)	1.5 (1.8)
Pneumonia	1.7 (1.9)	3.0 (3.1)	4.7 (3.8)	1.9 (4.2)	6.4 (5.2)	3.1 (1.8)	0.3 (0.4)
Total prescription rates macrolides by country	87.7	95.9	123.2	44.9	123.5	169.3	84.5

**Table 2:** Distribution of the prescription rates (×1000) of AMC by diagnostic by country and the 10 most frequently assigned diagnostic categories in 2010

Prescription rates (%)	France	Germany	Italy	Netherlands	Poland	Spain	United Kingdom
Bronchitis	15.2 (20.2)	1.8 (15.3)	14.1 (10.1)	3.2 (6.6)	15.0 (13.7)	17.0 (7.1)	1.2 (2.7)
Pharyngitis	8.9 (11.9)	0.2 (1.7)	17.4 (12.4)	0.3 (0.6)	15.7 (14.4)	8.0 (3.3)	0.2 (0.4)
Gastrointestinal infections	2.7 (3.6)	0.3 (2.5)	20.1 (14.4)	1.7 (3.5)	1.3 (1.2)	43.3 (18.0)	1.3 (2.9)
Other upper respiratory tract infections	2.4 (3.2)	0.4 (3.4)	8.8 (6.3)	1.4 (2.9)	6.0 (5.5)	12.8 (5.3)	1.2 (2.7)
Tonsillitis	0.6 (0.8)	1.1 (9.3)	20.6 (14.7)	1.6 (3.3)	18.2 (16.7)	35.8 (14.9)	0.3 (0.7)
Sinusitis	8.4 (11.2)	0.9 (7.6)	3.0 (2.1)	3.2 (6.6)	7.9 (7.2)	6.0 (2.5)	0.7 (1.6)
Skin infections	2.6 (3.5)	1.8 (15.3)	8.1 (5.8)	8.1 (16.8)	3.5 (3.2)	15.4 (6.4)	4.1 (9.1)
Cold/influenza	3.1 (4.1)	0.03 (0.3)	1.2 (0.9)	0 (0)	2.6 (2.4)	2.8 (1.2)	0.3 (0.7)
Otitis media	13.9 (18.5)	0.7 (5.9)	11.1 (7.9)	2.6 (5.4)	10.9 (10.0)	24.6 (10.2)	0.8 (1.8)
Pneumonia	2.5 (3.3)	0.8 (6.8)	1.5 (1.1)	4.7 (9.7)	2.7 (2.5)	5.7 (2.4)	0.2 (0.4)
Total prescription rates AMC by country	75.1	11.8	139.9	48.3	109.3	240.2	45.1

## Discussion

This study presented the total consumption of macrolides, lincosamides and AMC from IMS Health. It showed a variability of 12.1, 25, and >100-fold for macrolides, lincosamides and AMC, respectively, between countries. These antibacterials were mainly consumed in the ambulatory setting. This inter-country variability did extend to the diagnoses leading to their prescription.

The variability in the consumption of macrolides and AMC found in this study could be explained by the differences that have already been described among the European countries. The South, East and North Europe have been described as high, mild, and low antibacterial consumption countries, respectively (Elseviers et al. 2007). Italy, Poland, and the UK had the highest consumption of macrolides. Similarly, France, Italy, and Spain had the highest consumption of AMC.

Erythromycin, clarithromycin, and azithromycin were the most-used antibiotics in this study across all countries, between 2007 and 2010. The consumption of macrolides shifted from erythromycin to azithromycin, whereas the consumption of clarithromycin remained stable, and overall, there was a downward trend in the use of macrolides. Our results reproduced the results that were already described by Cars et al. in 1997 (Cars et al. 2001) using the same data provider and confirmed by the European Surveillance of Antimicrobial consumption (ESAC)-Net report in 2010 (Muller et al. 2010). The trend of the increasing consumption of AMC was consistent with the results from the ESAC-Net database, except for Spain, where the ESAC-Net database showed an increase in AMC consumption.

We explored the drug data source for potential explanations for the inter-country variability in drug consumption. The figures corresponding to ambulatory care were wholesalers’ sales in all countries, except in the Netherlands where they corresponded to the dispensed medicines. The figures in hospital consumption were wholesalers’ sales in Denmark, Norway, Poland, Sweden, and The Netherlands, whereas in the other five countries, they corresponded to the dispensed medications.

Problems with population coverage, which includes parallel trade, problems with drug data coverage and problems with a mix in the registration of antibiotics as ambulatory or hospital healthcare setting have been described to affect the validity of data when conducting cross-national comparison studies (Vander Stichele et al. 2004).

Beyond the fact that we included ambulatory and hospital healthcare settings, differences in the drug data source could explain the higher volume of consumption that the IMS data showed in our study compared with that of the ESAC-Net database. In the case of antibacterial drugs and countries such as Spain and Italy, wholesalers’ sales might provide a better estimation of the true exposed population to antibiotics, where approximately 30 % of all antibiotics are sold over-the-counter (Gagliotti et al. 2009; Llor et al. 2009). Conversely, the inclusion of parallel trade in the wholesalers’ sales could overestimate the antibiotic consumption in Spain, France, and Italy, countries that are known to be traditionally parallel exporters of pharmaceuticals (Panavos and Costa-Font 2005).

Other factors impact antibiotic consumption, including the number of different brand names (Monnet et al. 2005), availability of rapid diagnostic tests for upper respiratory tract infections (Cals et al. 2009), cultural differences (Deschepper et al. 2008), and differences in healthcare systems (Blommaert et al. 2014).

Spain, Italy, France, and Poland with high consumption of antibiotics, were also the countries with highest prescription rates. ICD-10 codes were taken as a proxy for indication for use. Although respiratory infections were the main diagnostic group assigned to the prescription of macrolides and AMC in all countries but United Kingdom, it showed a wide inter-country variability. Countries with higher consumption of antibiotics were also the countries with the higher percentage of these antibiotics prescribed for respiratory infections. A detailed look at specific diagnoses categories showed that macrolides and AMC are still prescribed for respiratory infections for which an antibiotic is not indicated, such as bronchitis or viral upper respiratory tract infections. This pattern of use of antibiotics for respiratory infections have been found also in the United States (Roumie et al. 2005; Shapiro et al. 2014), the Netherlands (van den Broek d’Obrenan et al. 2014) and United Kingdom (Petersen et al. 2007).

Another of the diagnostic categories often mentioned as the main reason for prescribing antibacterial drugs are genitourinary infections. In our study, none of the antibiotics included are first-choice empirical treatment of these infections, except amoxicillin-clavulanate in Spain (McQuiston Haslund et al. 2013). Our results showed that Spain was the country with the highest prescription rates of AMC for genitourinary infections.

The strengths of this study were, first, the inclusion of 10 European countries. Second, data on ambulatory and hospital healthcare settings were available at a population-based level. Third, the data came from a source different from the usual data sources to study antibiotic consumption in Europe, providing a complementary insight to what has been published up to now. Fourth, the use of a standard classification and a common unit of measurement eased the cross-national comparison of antibacterials utilisation. In addition to the DIDs, information on the consumption of antibiotics was provided as prescription rates. Finally, for this study information on the indication for use was available, raising once again the hypothesis of the potential misuse of antibiotics when comparing the results across countries.

One of the limitations of our study was the presentation of data for 2007–2010, as some prescribing patterns may have changed since then. ICD-10 codes, which were aggregated at level 3 might have introduced misclassification in the different diagnostic groups, as if any of the codes at level 4 included an infection as potential cause, the diagnostic code was classified in the corresponding group of infections. More granular diagnosis data could have provided a better insight of the real reason underlying each prescription. In addition, there were ICD-10 codes grouped under the other diagnoses group, which included a miscellany of diagnostic codes whose primary purpose was not clear. DIDs are used as a proxy of the number of people exposed to a medication when it is used chronically and only for a single indication, assuming that one DDD corresponds to the prescribed daily dose. However, antibiotics are used over short periods of time and intermittently; thus, this is a limitation of using the DIDs in our study. A more appropriate measure of the prevalence of exposure to antibiotics would be DDDs/1000 inhabitants/year (Capellà 1992). Nonetheless, all publications using the ATC/DDD methodology expressed antibiotic consumption in DIDs. Finally, not knowing the overall prevalence of use of all antibiotics did not allow us to calculate the percentage of use of macrolides, lincosamides and streptogramins, and AMC, out of the total antibiotic use in each of the countries.

## Conclusions

In conclusion, there is a wide variability in the consumption of macrolides and AMC across countries. Along with the distribution of the prescription rates by diagnoses groups, they reflect differences in the appropriateness of use of these antibiotics by country.

## Methods

Antibiotic consumption data were retrieved from the Multi-National Integrated Data Analysis System (MIDAS, IMS Health). It is a commercial database, which collects information on the sales of medicines from wholesalers or manufacturers to retail or hospital pharmacies. Data is sampled and projected to estimate sales for all retail and hospital pharmacies in the country. Data are registered per drug and all its dosages, and classified according to the Anatomical Classification of Pharmaceutical Products of the European Pharmaceutical Marketing Research Association (EphMRA). A booklet with the equivalences between the EphMRA classification and the Anatomical Therapeutic Chemical Classification (ATC) of the World Health Organization (WHO) (European Pharmaceutical Market Research 2013) allowed the classification of antibiotics for this study according to the 2013 version of the WHO ATC classification (WHO Collaborating Centre for Drug Statistics and Methodology 2015). The volume of consumption was expressed in defined daily doses (DDDs) per 1000 inhabitants and per day (DIDs). Population denominators were extracted from the official national statistics websites, reflecting the population at the end of the year.

Hospital and ambulatory consumption of macrolides (J01FA), lincosamides (J01FF), streptogramins (J01FG), and AMC (J01CR02) were analysed. The use of these antibacterials was assessed in Denmark, France, Germany, Italy, Norway, Poland, Spain, Sweden, the Netherlands and the United Kingdom between 2007 and 2010.

The selection of these antibiotics are part of the PROTECT project goals and conform to criteria described elsewhere (Abbing-Karahagopian et al. 2014). In summary, five key drug-adverse event pairs were selected fulfilling a set of a priori defined criteria that considered the public health impact of the adverse event and the possibility of studying a wide range of methodological aspects in pharmacoepidemiology. We categorised macrolides in erythromycin, clarithromycin, azithromycin, and other macrolides. The other macrolides group showed the widest variation in data availability between countries according to the different marketing authorisations across the countries. Clindamycin and lincomycin were approved in France, Germany, Italy, Poland, and Spain. Clindamycin was only approved in Denmark, the Netherlands, Norway, Sweden and the UK. For the streptogramins group, only quinupristin/dalfopristin was approved in France, Germany, Italy, Netherlands, Norway, Spain and the UK at the time of the study. For Denmark and Sweden only the combined in- and outpatient consumption data were provided. Between 2008 and 2010, data on the number of prescriptions sorted by diagnosis according to the International Classification of Diseases, 10th revision (ICD-10) (International Classification of Diseases, 10th revision 2015) were available for seven countries. IMS Health provided all physicians’ diagnosis and the treatment prescribed aggregated at country level. The diagnostic codes were given at three character-categories. We classified them into 45 categories, which were further assembled into four main groups: “respiratory infections”, “genitourinary infections”, “other infections”, and “other diagnoses”. Respiratory infections included the diagnostic codes for cold/influenza, pharyngitis, tonsillitis, laryngitis, sinusitis, otitis media, other diseases of the ear, bronchitis, pneumonia, unspecified respiratory disorders and other upper and lower respiratory tract infections. Genitourinary infections included upper- and lower urinary infections, male and female genital infections and other unspecified genito-urinary infections. The group other infections included different infections by anatomic organ as specified in the ICD classification and other bacterial, viral, protozoal and helminthic infections. The other diagnosis group refers to any diagnostic code not suggestive of an infection disease and it includes the rest of diseases included in the ICD classification. The prescription rate (×1000 inhabitants and year), by country, drug, and diagnosis group was estimated. As in the calculation of DIDs, population denominators were retrieved from the official national statistics websites.

The sources of drug consumption data were explored as sources of variation in drug consumption across the countries. See Table 3.

**Table 3:** Sources of drug consumption data by healthcare setting and country

Country	Sources of drug consumption data by healthcare setting	
	Outpatient setting	Inpatient setting
Denmark	Wholesalers’ sales combined out- and inpatient setting	
France	Wholesalers’ sales	Dispensed
Germany	Wholesalers’ sales	Dispensed
Italy	Wholesalers’ sales	Dispensed
Norway	Wholesalers’ sales	Wholesalers’ sales
Poland	Wholesalers’ sales	Wholesalers’ sales
Spain	Wholesalers’ sales	Dispensed
Sweden	Wholesalers’ sales combined out- and inpatient setting	
The Netherlands	Dispensed medicines	Wholesalers’ sales
United Kingdom	Wholesalers’ sales	Dispensed

All analyses were conducted using Microsoft Excel 2007^®^ (Microsoft Corporation, Redmond, WA, USA) and STATA13.1^®^ (StataCorpLP, College Station, TX, USA).

### Ethical approval

Not required.

## Additional files

10.1186/s40064-015-1398-4 Consumption of erythromycin, clarithromycin, azithromycin and amoxicillin-clavulanate in 10 European countries, 2007–2010 (expressed in defined daily doses per 1,000 inhabitants and per day).
10.1186/s40064-015-1398-4 Prescription rates of macrolides, lincosamides and stretogramins; and amoxicillin-clavulanate by country, 2008–2010. Prescription rates of macrolides, lincosamides and streptogramins by country, 2008–2010.
10.1186/s40064-015-1398-4 Prescription rates of erythromycin, azithromycin, clarithromycin, and other macrolides by diagnostic categories and country, 2010.

## Abbreviations

ATC: anatomic therapeutical chemical classification
DDD: defined daily doses
DID: defined daily doses per 1000 inhabitants and per day
EphMRA: European Pharmaceutical Marketing Research Association
ICD-10: International Classification of Diseases, 10th revision
MLS: macrolides, lincosamides and streptogramins
UK: United Kingdom
WHO: World Health Organization
