# Supplementary figures and images for: Sales of macrolides, lincosamides, streptogramins, and amoxicillin/clavulanate in the in- and outpatient setting in 10 European countries, 2007–2010
Source: Springerplus. 2015 Oct 15;4:612. doi: 10.1186/s40064-015-1398-4 (PMC4628133; doi:10.1186/s40064-015-1398-4)

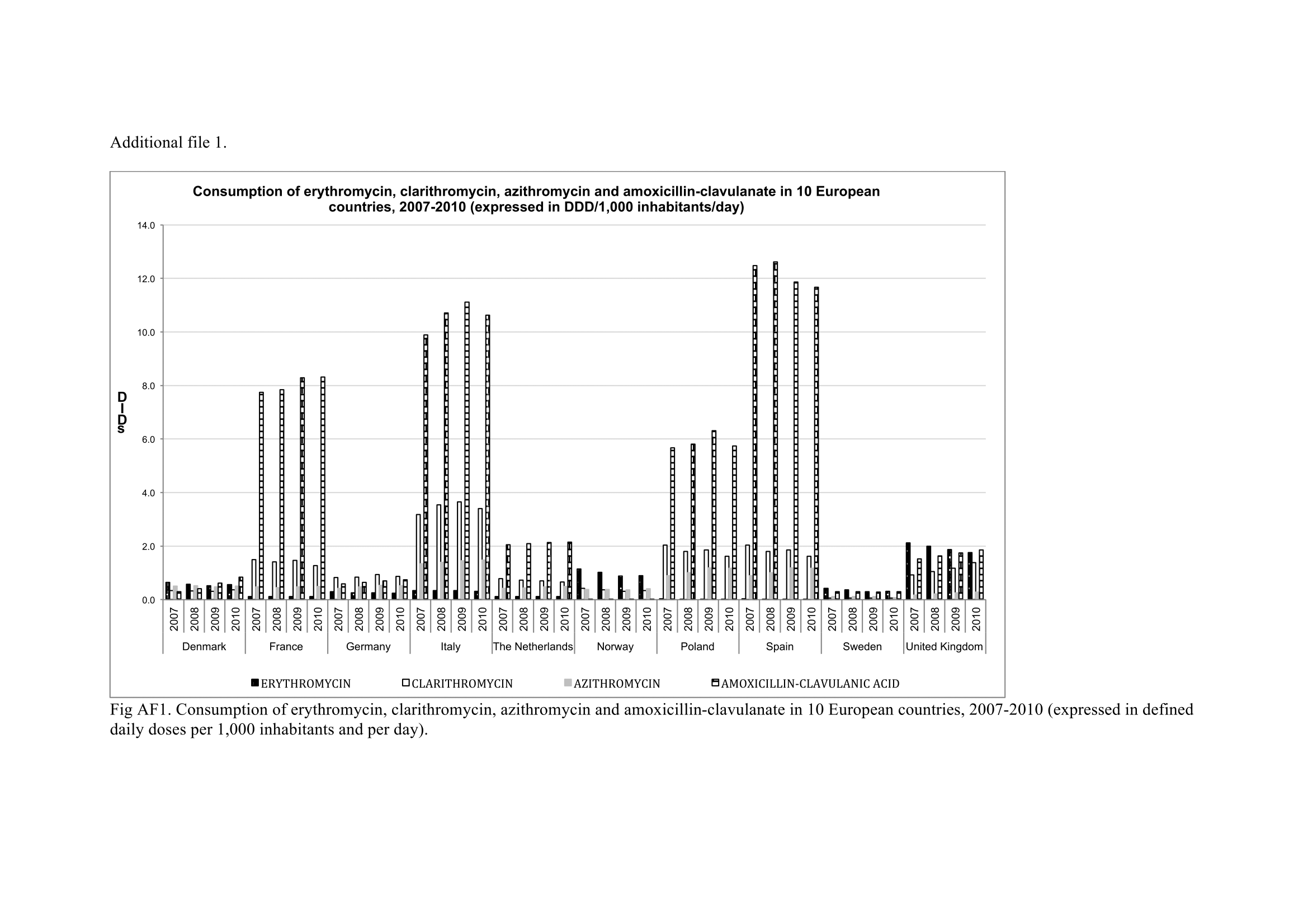

Supplement: Supplementary file 1 — 10.1186/s40064-015-1398-4 Consumption of erythromycin, clarithromycin, azithromycin and amoxicillin-clavulanate in 10 European countries, 2007–2010 (expressed in defined daily doses per 1,000 inhabitants and per day). [file 40064_2015_1398_MOESM1_ESM.png]

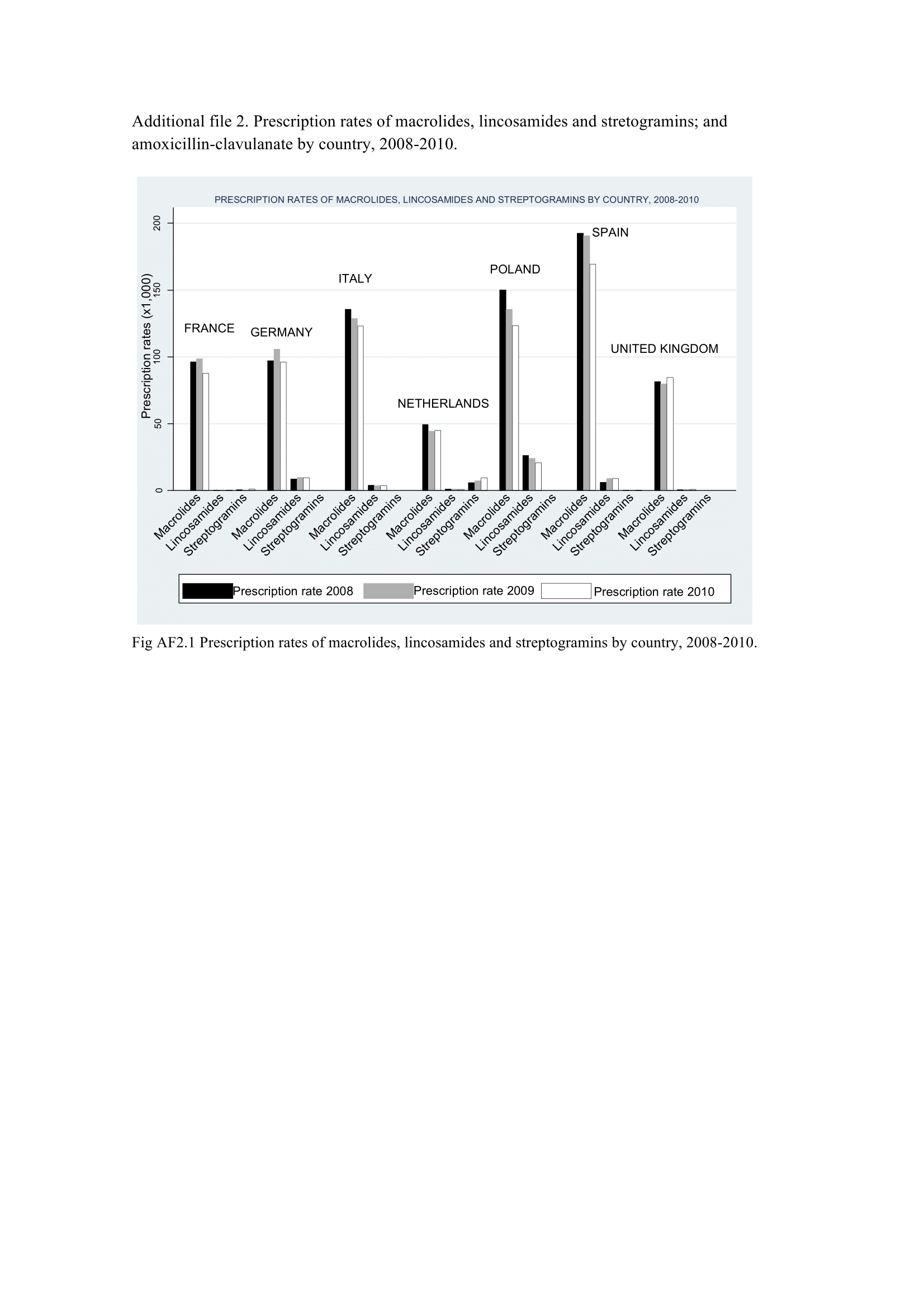

Supplement: Supplementary file 2 — 10.1186/s40064-015-1398-4 Prescription rates of macrolides, lincosamides and stretogramins; and amoxicillin-clavulanate by country, 2008–2010. Prescription rates of macrolides, lincosamides and streptogramins by country, 2008–2010. [file 40064_2015_1398_MOESM2_ESM.png]

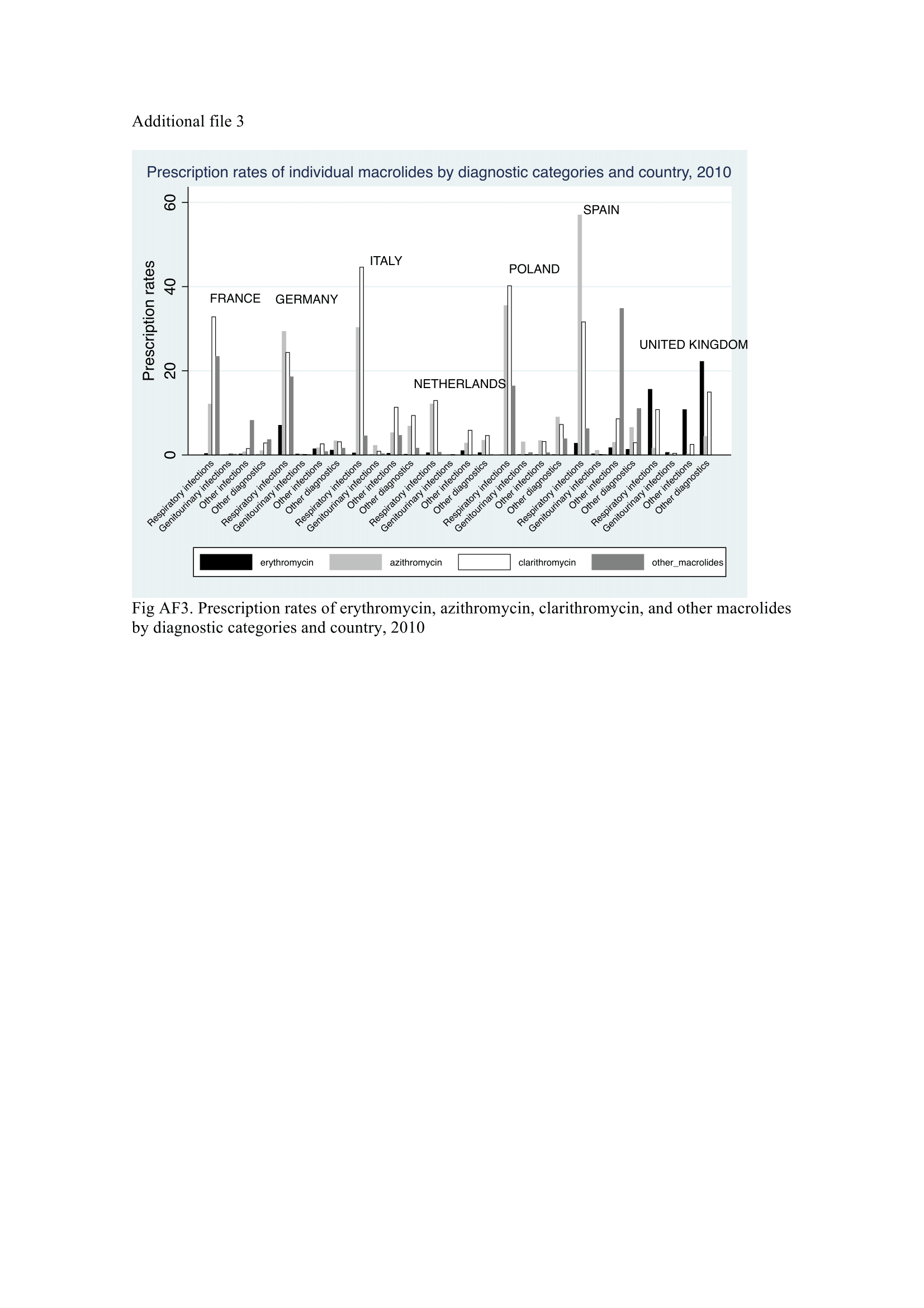

Supplement: Supplementary file 3 — 10.1186/s40064-015-1398-4 Prescription rates of erythromycin, azithromycin, clarithromycin, and other macrolides by diagnostic categories and country, 2010. [file 40064_2015_1398_MOESM3_ESM.png]
